# Pulmonary embolism complicated with necrotic debris in the lung parenchyma, treated by right lower lobectomy: a case report

**DOI:** 10.1186/s40792-018-0561-x

**Published:** 2019-01-09

**Authors:** Hikaru Watanabe, Naoki Kanauchi

**Affiliations:** Department of General Thoracic Surgery, Nihonkai General Hospital, 30, Akiho-cho, Sakata, Yamagata Japan

**Keywords:** Pulmonary infarction, Pulmonary embolism, Aseptic necrosis, Surgical treatment

## Abstract

**Background:**

It is uncommon for aseptic necrosis to occur in the lung parenchyma as a result of acute pulmonary embolism, because of the dual blood supply of the lung. We report a case in which acute pulmonary embolism led to pulmonary infarction and progressive lung necrosis, requiring treatment with right lower lobe resection.

**Case presentation:**

A 63-year-old man was referred to our hospital for right-side pleuritic chest pain and fever. He was then admitted in another department in our hospital with a diagnosis of pneumonia. Antimicrobial therapy was initiated; however, laboratory testing elevated white blood cell counts and C-reactive protein. Chest computed tomography revealed acute pulmonary emboli within the right lower lobe segmental pulmonary arteries, as well as a small cavity lesion within the same lobe, which measured 1.2 cm in diameter. Treatment strategies included anticoagulation therapy, thoracic drainage of the affected side, and an antibiotic escalation protocol. However, the patient’s fever did not subside; additionally, his leukocyte count increased after 3 days of the new treatment protocol. We considered it to be difficult to achieve cure with medical treatment alone; therefore, we performed a right lower lobectomy.

**Conclusions:**

We report an unusual case in which inflammation arose from lung necrosis secondary to lung embolism, which was not alleviated by conservative treatment; however, it was cured by right lower lobectomy.

## Background

It is uncommon for aseptic necrosis to occur in the lung parenchyma as a result of acute pulmonary embolism, due to the dual blood supply of the lung by both bronchial and pulmonary arteries. Necrosis of the lung due to pulmonary arterial obstruction is known as pulmonary infarction. Acute pulmonary embolism leads to pulmonary infarction in only 10% of cases [1]. In pulmonary parenchymal necrosis associated with pulmonary infarction, parenchymal fibrosis and reorganization occurs within 2 or 3 months after infarction; therefore, progressive necrosis of the pulmonary parenchyma is rare. We report a case in which acute pulmonary embolism led to pulmonary infarction and progressive lung necrosis, necessitating right lower lobe resection.

## Case presentation

**Fig. 1:**
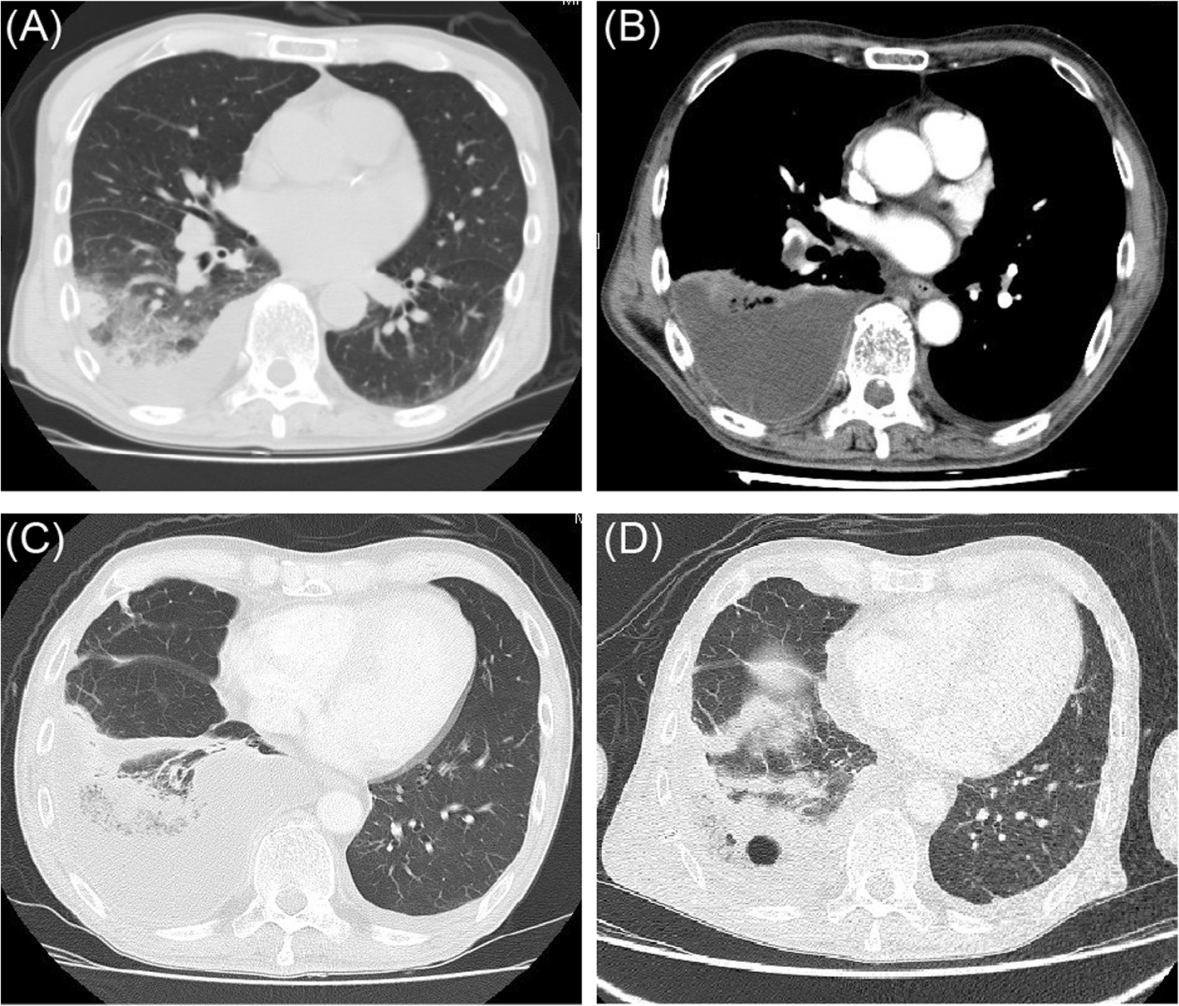
Axial sections on chest computed tomography (CT). **a** Plain CT at admission: infiltrative shadow in the right lower lobe and a small amount of right pleural effusion are visible. **b** Enhanced CT on post-admission day 7: an extensive lung embolism extending to the right basal pulmonary artery and A6 is seen. The pleural effusion had increased compared to that on initial CT. **c** Enhanced CT on post-admission day 7: persistent pulmonary infiltration is visible. **d** Enhanced CT on post-admission day 10: the pleural effusion decreased after thoracic drainage treatment. However, a cavitary lesion, 1.2 cm in maximum diameter, is visible in the infiltrative shadow in the right lower lobe

**Fig. 2:**
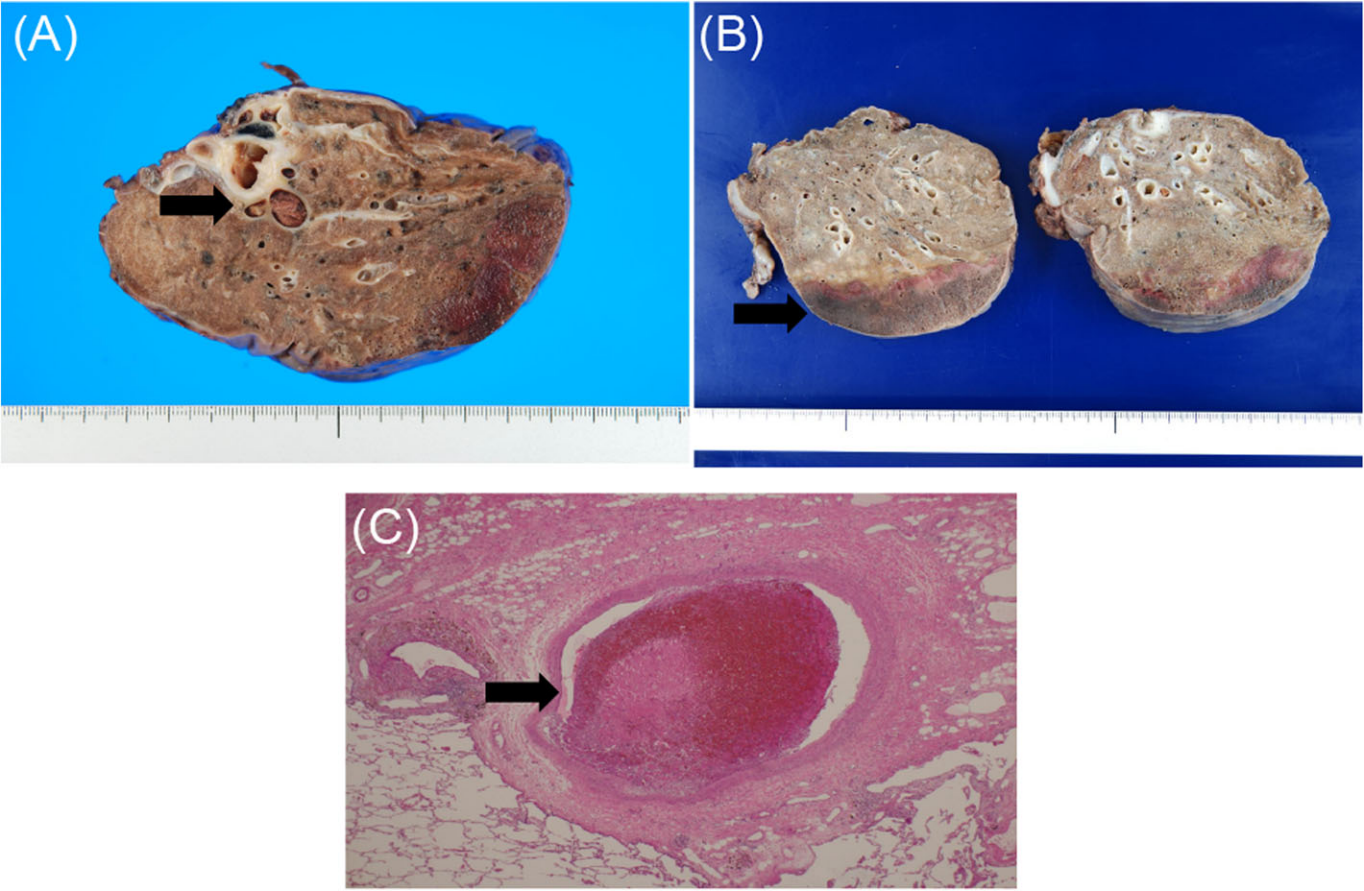
Histopathological findings. **a** Macroscopic findings: a light brown thrombus is visible in the pulmonary artery and dark red necrosis is visible in the peripheral pulmonary parenchyma. **b** Macroscopic findings: three layers of tissues (normal lung, intra-alveolar hemorrhage, and pulmonary necrosis layers) are visible from the proximal to the distal direction. **c** Microscopic findings: an organized thrombus is present in the pulmonary artery. Endothelial coverage, hyalinized fibrin, development of capillaries, and infiltration of fibroblasts are observed in the periphery of the thrombus, indicating its organization

A 63-year-old Japanese man without any medical history was referred to our hospital for right-sided pleuritic chest pain and fever. Laboratory investigations revealed a total leukocyte count of 7690/mm^3^ and serum C-reactive protein level of 17.8 mg/dL. Liver and kidney function tests were normal; sputum culture was negative. Chest computed tomography (CT) revealed a right basilar peripheral opacity and an ipsilateral reactive pleural effusion (Fig. 1a). The patient was treated for bacterial pneumonia with ceftriaxone (1.0 g twice per day), but experienced persistent fever of 39 °C until day 7 after admission. Therefore, contrast-enhanced CT was performed (Fig. 1b, c). Increased right pleural effusion and aggravated infiltration were observed, for which the patient was referred to our department (respiratory surgery). Contrast-enhanced CT showed a filling defect in the inferior lobar artery of the right lung, supporting a diagnosis of pulmonary embolism with reactive pleural effusion (Fig. 1b, c). The treatment strategies included anticoagulation therapy, thoracic drainage of the affected side, meropenem administration, and antibiotic protocol escalation. However, the fever did not subside; moreover, the leukocyte count increased to 15,300/mm^3^ by the third day after initiation of the new treatment (day 10 of admission). Therefore, contrast-enhanced CT was repeated, which revealed residual infiltration at the pulmonary embolism site in the right inferior lobe, with a cavity that measured 1.2 cm (Fig. 1d). Based on these findings, necrosis at the pulmonary parenchymal embolism site was considered progressive. Because we suspected that medical therapy alone would not be curative, we performed resection of the right lower lobe containing the embolism site. Pathology analysis showed a light brown thrombus in the pulmonary artery, with septic necrosis only in the peripheral parenchyma of the infarcted lung (Fig. 2a–c). Macroscopic examination in this case revealed three layers of tissues (proximal to distal): normal lung, intra-alveolar hemorrhage, and pulmonary necrosis (Fig. 2b). Postoperatively, meropenem was administered for 8 days; a new oral anticoagulant was administered for 3 months beginning on postoperative day 1 (edoxaban 30 mg/day). The postoperative course was uneventful; the patient has remained alive and disease-free for 18 months.

## Discussion

Clinically symptomatic pulmonary infarction comprises approximately 10% of all pulmonary embolisms [1]. Such infarction is regarded as related to occlusion of the peripheral pulmonary artery for the following reasons: (1) it is associated with increased venous pressure due to occlusive changes in the pulmonary vein, (2) blood flow can be observed from the bronchial artery to the lung, and (3) oxygen is supplied from the alveolar space.

The mechanism of pulmonary infarction involves occlusion of the pulmonary artery, which increases blood flow from the bronchial artery, thereby generating pressure beyond the capacity of the peripheral vascular bed and causing extravasation of red blood cells, which results in hemorrhagic infarction [2, 3]. Pathological changes in pulmonary infarction initially include hemorrhage in the alveolar space, followed by necrosis of the pulmonary parenchyma, which leads to progressive fibrosis and parenchymal reorganization. Histological examination in this case revealed three layers of tissues (proximal to distal): normal lung, intra-alveolar hemorrhage, and pulmonary necrosis. This finding was consistent with the progression of the pathology of pulmonary infarction. Horizontal layers with various pathological changes indicated that the pulmonary parenchyma received blood supply both from the pulmonary artery and the bronchial artery. In the present case, there was no sign of bronchial artery infarction, suggesting that oxygen was supplied via permeation of the bronchial artery. Thus, aseptic necrosis occurred only in the peripheral parenchyma of the infarcted lung.

Thus far, there remains insufficient information regarding aseptic lung necrosis secondary to lung infarction; however, formation of an aseptic cavity has been reported in association with lung infarction. Risk factors for pulmonary infarction with cavitation include a sizable area of consolidation (> 4 cm), older age, a history of heart failure, and a history of chronic lung disease [4, 5]. The present patient did not show any accompanying disease, but had a lung infarction lesion of ≥ 4 cm. Infected pulmonary infarctions lead to cavitation more rapidly than bland infarctions with aseptic necrosis. The mean time to cavitation for an infected pulmonary infarction is 18 days [4]. If the patient had been followed up without treatment for another 1 week, infection might have occurred at the site of the pulmonary infarction. Furthermore, analysis of a large autopsy series has shown that cavity formation in the lung is uncommon in cases of infarction: such cavities are found in only 4–7% of all pulmonary emboli [5]. The lung cavity may result from either secondary infection of the infarcted tissue or sterile necrosis. The infarct is typically aseptic and cavitation is induced by infection, which leads to abscess formation. However, the patient in the present case exhibited aseptic cavitation after a sterile pulmonary infarction.

We anticipated bronchial stump fistula and empyema as serious postoperative complications in the present case. To prevent such complications, it is important to secure blood flow to the bronchial stump and control infection. The reported incidence of bronchial stump fistula associated with lung lobectomy for lung cancer is 0.5–4% [6]. Notably, right lower lobectomy exhibits an increased risk of bronchial stump fistula [7]. This is likely due to impaired bronchial arterial circulation to the bronchial stump of the right lower lobe. In the patient in the present case, lymph node dissection was not required, unlike conventional surgery for lung cancer; thus, there was no risk of injury to the bronchial artery, and blood flow to the bronchial fistula was secured in an uncomplicated manner.

## Conclusions

In summary, we have reported a case in which lung inflammation arose from lung necrosis secondary to pulmonary embolism, which was not alleviated by conservative treatment; however, it was cured by right lower lobectomy.

## Abbreviations

CT: Computed tomography
